# Bioactive Compound Profiling of Agarophyte Seaweed (*Gelidiella acerosa*, *Gracilaria arcuata*, and *Gracilaria verrucosa*) Based on LC-HRMS Metabolomic and Molecular Networking Approach

**DOI:** 10.3390/foods14234042

**Published:** 2025-11-25

**Authors:** Maria Dyah Nur Meinita, Riyanti, Dyahruri Sanjayasari, Riviani, Dicky Harwanto, Apisada Jiso, Till F. Schäberle, Ute Mettal, Il-Soo Moon, Jae-Suk Choi

**Affiliations:** 1 Faculty of Fisheries and Marine Science, Jenderal Soedirman University, Purwokerto 53123, Indonesia; maria.meinita@unsoed.ac.id (M.D.N.M.);; 2 Agricultural Biotechnology Magister Program, Graduate School, Jenderal Soedirman University, Purwokerto 53123, Indonesia; 3 Faculty of Fisheries and Marine Science, Diponegoro University, Semarang 50275, Indonesia; 4 Department of Pharmaceutical Sciences, Faculty of Pharmacy, Chiang Mai University, Chiang Mai 50200, Thailand; 5 Faculty of Agricultural Sciences, Nutritional Sciences and Environmental Management, Justus Liebig University, 35390 Giessen, Germany; 6 Department of Anatomy, College of Medicine, Dongguk University, Gyeongju 38066, Republic of Korea; 7 Department of Seafood Science and Technology, The Institute of Marine Industry, Gyeongsang National University, Tongyeong-si 53064, Republic of Korea

**Keywords:** seaweed, macroalgae, bioactive, pharmacological, *Gelidiella*, *Gracilaria*

## Abstract

To date, exploration of *Gracilaria* and *Gelidiella*’s bioactive compounds has been conducted using conventional methods that require a long time, high costs, and significant effort. Currently, metabolomic profiling and molecular networking have emerged as methods of exploring bioactive compounds. This study aimed to perform bioactive compound profiling through a metabolomic LC-HRMS-based and molecular networking approach in *Gelidiella acerosa*, *Gracilaria arcuata*, and *Gracilaria verrucosa*. All chromatograms and MS/MS spectra obtained for three crude extracts were digitally converted into an mzXML file using MSConvert, submitted to the Global Natural Product Social (GNPS), and visualized in Cytoscape 3.9.1. In total, nine dereplicated compounds were identified: 11-Deoxyprostaglandin (*m*/*z* 324.214), Diacylglyceryl trimethylhomoserines (DGTS) (*m*/*z* 684.575), Glycochenodeoxy acid (*m*/*z* 448.369), Lysophosphatidylcholine (*m*/*z* 522.350), Diacylglyceryl trimethylhomoserines (DGTS) (*m*/*z* 656.557), Pheophorbide A (*m*/*z* 593.266), Pyropheophorbide A (*m*/*z* 593.266), (2R,3R)-2-(3,4-dihydroxyphenyl)-3,5,7-trihydroxy-2,3-dihydro-4H-chromen-4-one (*m*/*z* 303.15), and Polyporic acid (*m*/*z* 293.156). These compounds are typically classified as fatty acids, lipids, terpenoids, alkaloids, shikimates, and phenylpropanoids. The molecular networking and metabolite clustering showed an interesting pattern where some compounds were produced only by one species, some by two species, and some by all three. These compounds may have pharmaceutical potential based on their chemical properties and reported activities.

## 1. Introduction

Seaweed or marine macroalgae play an important role both ecologically and economically. According to Ref. [1], the world’s total alga production reached 38 million tonnes, an increase of 4% compared to the previous data in 2020. With a share of 97% of the world’s alga production, Asian countries has confirmed their status as leading producers of marine macroalgae. In 2022, China accounted for 60% of entire production, followed by Indonesia (25%) and the Republic of Korea (5%). Red seaweed (Rhodophyta) is one of the most abundant seaweeds among brown seaweed and green seaweed. Agar-producing seaweed (agarophyte) species of the genus *Gracilaria*, *Gelidium*, *Gelidiella,* and *Pterocladia* are among the economically important algae and account for world agar production. *Gracilaria* and *Gelidium* account for 53% and 44% of the world’s agar production, respectively, whereas other agarophytes, such as *Gelidiella* and *Pterocladia* account for 3% [2,3,4].

Besides their agar content, agarophytes are rich in primary and secondary metabolites. In the harsh environment of marine life, including desiccation, salinity, radiation, temperature, and nutrients, seaweeds produce a unique bioactive compound that is distinct from bioactive compounds from terrestrial environments [5,6,7]. Seaweeds represent one of the first marine creatures to be chemically examined and have become a source of novel bioactive substances. Over 3600 publications have described 3300 secondary metabolites from marine plants and algae, making them an almost limitless source of novel bioactive substances [8,9]. To date, metabolite studies of agarophytes have been conducted using conventional methods that require a long time, high costs, and significant effort. Nowadays, the rapid development of high-throughput techniques, biotechnology instruments, and bioinformatic tools helps us to explore agarophytes’ metabolites more efficiently in a shorter time [8,10,11].

Compared to traditional bioassay-guided methods, liquid chromatography–high resolution mass spectrometry (LC-HRMS)-based metabolomics is very useful in revealing the undiscovered chemical diversity of seaweed. Metabolomics, as a newer emerging discipline within “omics”, can be used to analyze the metabolites of agarophytes qualitatively and quantitatively and is becoming an essential tool for rapid and accurate analysis of high-throughput data to find new information on various metabolites generated by seaweed [12]. LC-HRMS, combined with NMR and chemometrics, is now the standard workflow for profiling untargeted metabolites in seaweed species. This method allows rapid dereplication, compound class annotation, and correlation with bioactivity. However, untargeted LC-HRMS-based metabolomics studies specifically for the genera *Gracilaria* and *Gelidiella* are relatively rare. Several recent studies of the genus *Gracilaria* have used LC-HRMS methods to analyze untargeted metabolites from *Gracilaria edulis*, *Gracilaria foliifera*, and *Gracilaria debilis* [13,14,15]. For the genus *Gelidiella*, several studies have been conducted using LC-MS for targeted compounds, but no LC-HRMS-based metabolomics studies for non-targeted compounds have been performed [16,17].

Members of the genera *Gelidiella* and *Gracilaria* are found from tropical to temperate regions and have long been utilized as an agar source, but most of their potential for pharmaceutical, nutraceutical, and therapeutic agents has remained unexplored [18]. In this study, we tried to perform a comprehensive analysis of three different *Gelidiella* and *Gracilaria* species, *Gelidiella acerosa*, *Gracilaria arcuata*, and *Gracilaria verrucosa*, to gather information on their bioactive compounds based on LC-HRMS metabolomic profile and molecular networking and their potential application. Hence, a comprehensive study of metabolite profiling from agarophytes is needed to develop a better understanding of their primary and secondary metabolite functional importance and wide range of further applications as pharmaceutical, nutraceutical, and therapeutic agents.

## 2. Materials and Methods

The methodology of the metabolomic profiling and molecular networking of *Gelidiella acerosa*, *Gracilaria arcuata*, and *Gracilaria verrucosa* is shown in Supplementary Figure S1.

### 2.1. Sampling Site

**Figure 1:**
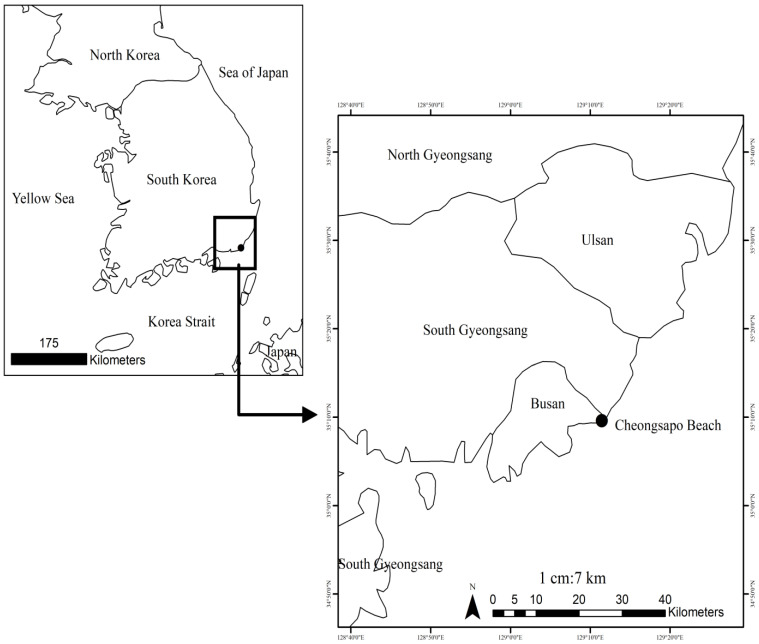
Sampling site and study area.

*Gelidiella acerosa*, *Gracilaria arcuata*, and *Gracilaria verrucosa* were collected from Cheongsapo, Busan, South Korea (35°9′35.14″ N; 129°11′27.64″ E) (Figure 1). The sampling site is located on the southern coast of the Korean Peninsula, with warm current influence and substrates that create relatively favorable conditions for red seaweed. Thirty fronds of each red seaweed sample were collected randomly. The red seaweed samples were transported to the laboratory, washed thoroughly with distilled water to remove salts, sand, and epiphytes, and then air-dried at room temperature (30–35 °C).

### 2.2. Morphological and Molecular Identification

The identification of *G. acerosa*, *G. arcuata*, and *G. verrucosa* was confirmed based on morphological and anatomical characteristics. Morphological features were examined under a microscope and documented photographically. Morphological and anatomical identification was performed using the following method of [19]. The voucher specimens were kept at the Seaweed Biotechnology Laboratory of Prof. Hong Yong Ki, Pukyong National University, South Korea.

### 2.3. Pretreatment

The clean seaweed samples were air-dried completely for 1 week at room temperature (30–35 °C). The dried materials were then ground into fine powder using an electric grinder for 10 min and stored in vials until extraction.

### 2.4. Extraction of Bioactive Compounds

Extraction was performed using the maceration method. Approximately 1 g of dried and powdered *G. acerosa*, *G. arcuata*, and *G. verrucosa* was extracted with 50 mL of methanol (1:50, *w*/*v*) for 48 h at room temperature with continuous shaking. The extracts were filtered, and a rotary evaporator was applied to evaporate the solvent. The crude extracts were subsequently preserved at −20 °C for further use.

### 2.5. LC–HRMS Analysis

LC-HRMS measurements were carried out at the Natural Products Laboratory of Justus Liebig University Giessen, Germany, by analyzing the extract at a final concentration of 1 mg/mL. LC-HRMS measurements were performed using a Micro-TOF-Q II mass spectrometer (Bruker, Billerica, MA, USA) with an ESI source combined using a Dionex Ultimate 3000 HPLC (Thermo Scientific, Darmstadt, Germany) with an EC10/2 Nucleoshell C18 2.7 column μm (Macherey–Nagel, Düren, Germany) at 25 °C with an injected volume of 2 μL with methanol as a blank control [20]. The LC-HRMS system was operated in positive ion mode and a linear gradient elution was used, with mobile phases consisting of water with 0.1% formic acid (A) and methanol with 0.1% formic acid (B), flow: 600 µL/min (0 min: 95% A; 0.80 min: 95% A; 18.70 min: 4.75% A; 18.80 min: 0% A; 23.00 min: 0% A; 23.10 min: 95% A; 25.00 min: 95% A) [20].

### 2.6. MS-Based Molecular Networking

MS data were transformed from MassHunter files (.d) into mzXML format using MS Convert version 3 [20]. Next, the converted files were then analyzed through the Global Natural Product Social Molecular Networking (GNPS; gnps.ucsd.edu.com) platform and visualized in Cytoscape 3.9.1 software [21]. The processing of LC-HRMS data was analyzed descriptively, where the variables used were differentiated based on the differences between species. The LC-HRMS profiling provides insights into the molecular mass, structural characteristics, identification, and relative abundance of the compounds present in the sample. The results of the analysis of the LC- HRMS data yielded a peak high-flow chromatogram that allowed us to determine the molecular weight of the compounds contained in the extract.

### 2.7. Data Analysis

MS data processing was carried out with DataAnalysis 4.4 (Bruker, Billerica, MA, USA), samples were organized based on clustering outcomes, and pairwise similarity values were utilized to classify them into metabolic groups. When two clustered samples exhibited a similarity score of 0.7 or higher, they were categorized within the same metabolic group and formed a network indicative of unique molecules and edge molecules linked with similar molecules. To visualize the distribution of the compound, node colors were assigned based on sample collection. Dereplication was carried out by automatically comparing the GNPS spectral libraries with the experimental MS/MS spectra. A cosine score > 0.7 and low parent mass error (<10 ppm) were used to classify annotations as confident matches.

### 2.8. Feature Annotation

Detected molecular features were annotated by comparing exact mass and fragmentation patterns with reference spectra from GNPS. Unknown features were classified as unidentified compounds for further study.

## 3. Results and Discussion

**Figure 2:**
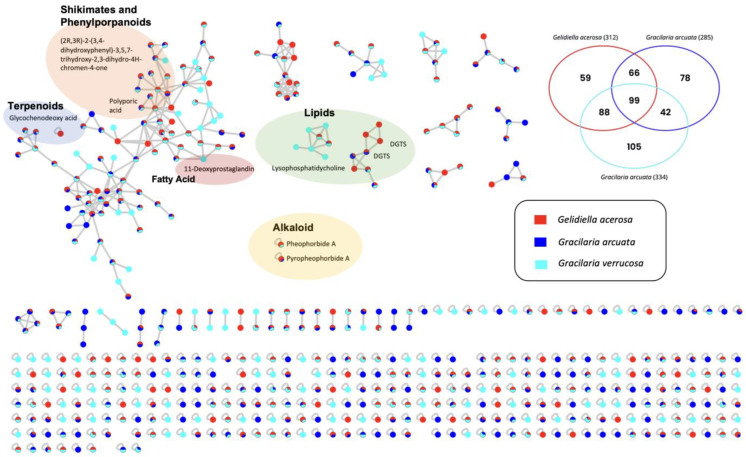
Molecular network observed in three species of macroalgae. Node colors are based on each species of macroalga. Red: *Gelidiella acerosa*; dark blue: *Gracilaria arcuata*; and light blue: *Gracilaria verrucosa*. Nodes highlighted in the colored box represent parent ions that were derived as Glycochenodeoxy acid (*m*/*z* 448.369), (2R,3R)-2-(3,4-dihydroxyphenyl)-3,5,7-trihydroxy-2,3-dihydro-4H-chromen-4-one (*m*/*z* 303.15), Polyporic acid (*m*/*z* 293.156), 11-Deoxyprostaglandin (*m*/*z* 324.214), Lysophosphatidylcholine (*m*/*z* 522.350), Diacylglyceryl trimethylhomoserines (DGTS) (*m*/*z* 684.575), Diacylglyceryl trimethylhomoserines (DGTS) (*m*/*z* 656.557), Pheophorbide A (*m*/*z* 593.266), and Pyropheophorbide A (*m*/*z* 593.266).

The molecular network of metabolite mass spectrometry profiles from three types of agar-producing seaweed, namely, *Gracilaria verrucosa*, *Gelidiella acerosa*, and *Gracilaria arcuata*, was visualized based on differences in seaweed species. Identification of bioactive metabolites in seaweed extract was carried out using LC-HRMS analysis. This analysis leads to the characterization of dereplicated and non-dereplicated compounds using GNPS (The Global Natural Product Social Molecular Networking) analysis and visualized using Cytoscape 3.9.1 software. In total, 537 metabolites were identified (13 in positive node, 524 in negative node) from extracts of three seaweeds species that produce different secondary metabolites (alkaloids, fatty acids, terpenoids, and polyketides) (Figure 2).

**Figure 3:**
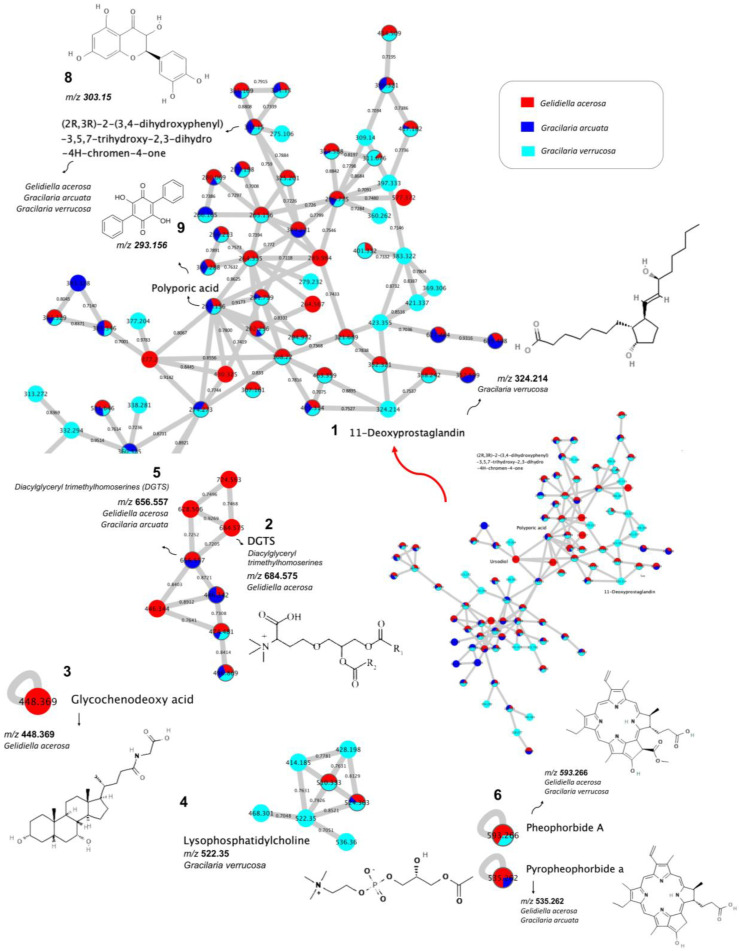
Chemical structures of the dereplicated compounds of *Gracilaria verrucosa*, *Gelidiella acerosa*, and *Gracilaria arcuata:* (1) 11-Deoxyprostaglandin (*m*/*z* 324.214), (2) Diacylglyceryl trimethylhomoserines (DGTS) (*m*/*z* 684.575), (3) Glycochenodeoxy acid (*m*/*z* 448.369), (4) Lysophosphatidylcholine (*m*/*z* 522.350), (5) Diacylglyceryl trimethylhomoserines (DGTS) (*m*/*z* 656.557), (6) Pheophorbide A (*m*/*z* 593.266), (7) Pyropheophorbide A (*m*/*z* 593.266), (8) (2R,3R)-2-(3,4-dihydroxyphenyl)-3,5,7-trihydroxy-2,3-dihydro-4H-chromen-4-one (*m*/*z* 303.15), and (9) Polyporic acid (*m*/*z* 293.156. Node colors are based on each species of macroalga. Red: *Gelidiella acerosa*; dark blue: *Gracilaria arcuata*; and light blue: *Gracilaria verrucosa*.

Based on the compounds that have been dereplicated, there are three molecular networks containing dereplicated compounds, and the other six compounds are single nodes, which means they do not have a network with other compounds (Figure 3). Compounds of 11-Deoxyprostaglandin (*m*/*z* 324.214), (2R,3R)-2-(3,4-dihydroxyphenyl)-3,5,7-trihydroxy-2,3-dihydro-4H -chromen-4-one (*m*/*z* 303.15), and polyporic acid (*m*/*z* 293.156) are present in one molecular network.

**Figure 4:**
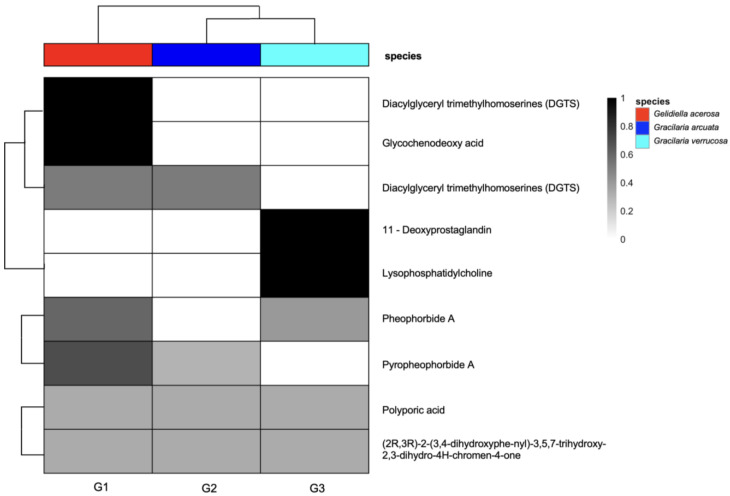
Heatmap and clustering of secondary metabolites detected from *Gelidiella acerosa*, *Gracilaria arcuata*, and *Gracilaria verrucosa*. The color gradient represents the relative abundance of each compound (black = high; white = low).

To provide an overview of the metabolite composition among the three agarophyte species through molecular networking, a heatmap of the LC-HRMS data was constructed (Figure 4).

The heatmap illustrates the relative abundance and presence–absence patterns of dereplicated compounds across *Gelidiella acerosa*, *Gracilaria arcuata*, and *Gracilaria verrucosa*. Distinct clustering of metabolites and species indicates differences in metabolite profiles among the three agarophytes. For instance, diacylglyceryl trimethylhomoserines (DGTSs), and glycochenodeoxy acid are predominantly detected in *G. acerosa*. In contrast, 11-deoxyprostaglandin and lysophosphatidylcholine are specific to *G. verrucosa*. This visualization highlights interspecific variation in secondary metabolite production and supports the subsequent molecular networking analysis.

Interestingly, molecular networking showed that four compounds were produced only by one species, while three compounds were produced by two species and two compounds were produced by three of them. The compound lysophosphatidylcholine (*m*/*z* 522,350) has its own molecular network with nodes worth *m*/*z* 468,301; 414,185; 428,198; 536.36 which was produced specifically from *Gracilaria verrucosa* and two other nodes with *m*/*z* values 510.333, 50% of which were produced from *Gracilaria verrucosa* and 50% from *Gelidiella acerosa*, and *m*/*z* 524.363, 50% of which were produced from *Gracilaria verrucosa,* 38% from *Gelidiella acerosa,* and 12% from *Gracilaria arcuata*. The diacylglyceryl trimethylhomoserine (DGTS) *m*/*z* 656.557 and *m*/*z* 684.575, both produced specifically by one type of seaweed, have a network with DGTSs produced by two types of seaweed (*Gelidiella acerosa* and *Gracilaria arcuata*). Meanwhile, glycochenodeoxy acid (*m*/*z* 448.369), pheophorbide a (*m*/*z* 593.266), and pyropheophorbide a (*m*/*z* 535.262) are compounds that do not have a network with other compounds. Seaweed species produce secondary metabolites in the form of compounds with known parent mass and adduct values. The eight dereplicated compounds produced specifically by one type of seaweed are shown in Table 1.

*Gracilaria verrucosa* produces two dereplicated compounds, namely, 11-Deoxyprostaglandin (*m*/*z* 324.214), and lysophosphatidylcholine (*m*/*z* 522.350). *Gelidiella verrucosa* produces 11-Deoxyprostaglandin that has potential as an anti-inflammatory drug. A chemical study on the anti-inflammatory components of the red alga *Gracilaria verrucosa* led to the isolation of new 11-deoxyprostaglandins [22]. A further four dereplicated compounds are known to be produced specifically by seaweed species. Compounds from *Gelidiella acerosa* include diacylglyceryl trimethylhomoserines (DGTSs) (*m*/*z* 684.575) and glycochenodeoxy acid (*m*/*z* 448.369). *Gelidiella acerosa* produces diacylglyceryl trimethylhomoserines (DGTSs), a lipid class reported to exhibit anti-inflammatory activity in related red seaweeds, which could potentially contribute to anti-inflammatory effects [23]. An increasing number of studies have shown that bioactive compounds from seaweeds may possess anti-inflammatory properties [24].

There are three compounds produced by two types of seaweed, including DGTS, pheophorbide a, and pyropheophorbide a (Table 2).

DGTS with a parent mass value of *m*/*z* 656.557 is produced from two types of seaweed, namely, *Gelidiella acerosa* and *Gracilaria arcuata*, which have potential in anti-inflammatory drugs. An increasing number of studies have reported that bioactive compounds found in seaweeds could have possible anti-inflammatory activities. Metabolites with anti-inflammatory properties were isolated from seaweeds such as phlorotannins, polyphenols, glycosterols, polysaccharides, and polyunsaturated fatty acids (PUFAs). Pheophorbide a was a compound with parent mass value *m*/*z* 593.266 produced from the seaweeds *Gelidiella acerosa* and *Gracilaria verrucosa* that has potential anti-inflammatory, anti-genotoxic [25], and anticancer activity [26]. Pyropheophorbide, a compound with a parent mass value of *m*/*z* 535.262, is produced from the seaweeds *Gelidiella acerosa* and *Gracilaria arcuata* and shows a pathway with alkaloids. There are two compounds produced by all three seaweeds, namely, (2R,3R)-2-(3,4-dihydroxyphenyl)-3,5,7-trihydroxy-2,3-dihydro-4H-chromen-4-one (*m*/*z* 303.15) and polyporic acid (*m*/*z* 293.156) (Table 3).

Dihydroquercetin (DHQ), a dihydroflavonol also known as taxifolin ((2R, 3R)-2-(3, 4-dihydroxyphenyl)- 3,5,7-trihydroxy-2,3-dihydro-4H-chromen-4-one) [27], has been reported to exert a wide range of biological activities, including antioxidant activity [28], lipid peroxidation inhibition [29], radical formation deceleration [30], antihemolytic, antiaggregatory, antitumor, hepatoprotective, anesthetic, and immunocorrecting properties [27,31]. Polyporic acid shows potential antifungal activity [32] against *L*. *plantarum*, *L*. *fermentum*, *L*. *brevis*, and *L*. *paracasei* caused by phenyllactic and polyporic acids, which are the product of phenylpyruvic acid. In the presence of phenylpyruvic acid, these *lactobacilli* strains exhibit higher antifungal activity than in previous studies. Compounds produced from *Gracilaria verrucosa*, *Gelidiella acerosa*, and *Gracilaria arcuata* were alkaloids, fatty acids, shikimates and phenylporpanoids, and terpenoids. *Gelidiella acerosa* seaweed produces the most dereplicated secondary metabolites compared to the other two types of seaweed. These compounds are reported in the literature to exhibit activities that may be of pharmaceutical interest [17,19,20,21,23,27].

Studies on untargeted LC-HRMS-based metabolomics in the genera *Gracilaria* and *Gelidiella* are still very limited. Two LC HRMS-based metabolomics studies were conducted by [13,14] to profile the chemical composition of methanol extracts from Gracilaria species (*G. edulis* and *G. folifera*). In the *G. edulis* study, the combination of HR-LCMS and 1H-NMR revealed a relatively wide diversity of secondary metabolites, including alkaloids, amino acids, aromatic compounds, benzoquinones, carotenoids, fatty acids, flavonoids, secondary alcohols, diterpenes, phenolics, sesquiterpenoids, and triterpenoids [13]. In the study of *G. folifera* using the LCHRMS metabolomics approach, several molecules were identified and evaluated against cancer cell lines [14]. Meanwhile, for the *Gelidiella* genus, metabolomics research conducted so far has used LC-MS/MS- and GC-MS-based methods. Research on bioactive components of *G. acerosa* using LC-MS/MS and GC-MS has identified compounds such as phytol and several other bioactive components that may contribute to the observed neuroprotective activity.

It has been demonstrated that metabolomics profiling and molecular networking of *G. acerosa*, *G. arcuata*, and *G. verrucosa* are potent and effective techniques for identifying metabolic responses and patterns. Bioassays are still required, nevertheless, in order to confirm the roles of the chemomarkers discovered. The development of ecological bioassays for metabolite function may also facilitate the identification of favorable environmental factors that stimulate metabolite production, which could have a direct impact on bioprospecting. The utilization of these bioactive compounds from these three agarophyte seaweed species in the nutraceutical and therapeutic industry presents significant challenges due to their stability, bioavailability, and compatibility with the processing conditions. Further exploration of seaweed bioactivity can provide innovative solutions for pharmaceutical, nutraceutical and therapeutic needs and biotechnology-based product development.

## 4. Conclusions

Promising future research directions include the integration of genomic, transcriptomics, proteomics, and metabolomics to uncover the biosynthetic pathways of active compounds [33,34]. Further studies on the isolation, purification, and fractionation of dereplicated compounds are required to bring agarophytes to pharmaceutical, nutraceutical, and therapeutic applications. Furthermore, standardization of extraction and analysis methods is still required to confirm the global equivalence of research results [35]. Further in vivo research and preclinical trials are also needed to confirm the pharmacological effects demonstrated in vitro and in the bioassay [36,37]. With research increasingly focused on green technology and bioeconomic valorization, the genera *Gracilaria* and *Gelidiella* hold great promise as a source of multifunctional biomolecules for nutraceutical and therapeutic applications.

## Figures and Tables

**Table 1:** Compounds produced by one species of seaweed (*Gracilaria verrucosa*, *Gelidiella acerosa*, and *Gracilaria arcuata*).

Compound Code	Compound	Class	Parent Mass (*m*/*z*)	Adduct	Source
[1]	11-Deoxyprostaglandin	Fatty Acids	324.214	M + H − H_2_O	*Gracilaria verrucosa*
[2]	Diacylglyceryl trimethylhomoserines (DGTSs)	Lipids	684.575	M + H	*Gelidiella acerosa*
[3]	Glycochenodeoxy acid	Terpenoids	448.369	M + H	*Gelidiella acerosa*
[4]	Lysophosphatidylcholine	Lipids	522.350	M + H	*Gracilaria verrucosa*

**Table 2:** Compounds produced by two species of seaweed (*Gracilaria verrucosa*, *Gelidiella acerosa*, and *Gracilaria arcuata*).

Compound Code	Compound	Class	Parent Mass (*m*/*z*)	Adduct	Source
[5]	Diacylglyceryl trimethylhomoserines (DGTS)	Lipids	656.557	M + H	*Gelidiella acerosa* *Gracilaria arcuata*
[6]	Pheophorbide A	Alkaloids	593.266	M + H	*Gelidiella acerosa* *Gracilaria verrucosa*
[7]	Pyropheophorbide A	Alkaloids	535.262	M + H	*Gelidiella acerosa* *Gracilaria arcuata*

**Table 3:** List of derived compounds produced by the three seaweed species (*Gracilaria verrucosa*, *Gelidiella acerosa*, and *Gracilaria arcuata*).

Compound Code	Compound	Class	Parent Mass (*m*/*z*)	Adduct	Source
[8]	(2R,3R)-2-(3,4-dihydroxyphenyl)-3,5,7-trihydroxy-2,3-dihydro-4H-chromen-4-one	Shikimates and Phenylpropanoids	303.15	M + H	*Gelidiella acerosa* *Gracilaria arcuata* *Gracilaria verrucosa*
[9]	Polyporic acid	Alkaloids	293.156	M + H	*Gelidiella acerosa* *Gracilaria arcuata* *Gracilaria verrucosa*

## Data Availability

The original contributions presented in the study are included in the article; further inquiries can be directed to the corresponding authors.

## Supplementary Materials

The following supporting information can be downloaded at: https://www.mdpi.com/article/10.3390/foods14234042/s1, Figure S1: The methodology of metabolomic profiling and molecular networking approach of *Gelidiella acerosa*, *Gracilaria arcuata*, and *Gracilaria verrucosa*.
