# Supplementary material for: Bioactive Compound Profiling of Agarophyte Seaweed (Gelidiella acerosa, Gracilaria arcuata, and Gracilaria verrucosa) Based on LC-HRMS Metabolomic and Molecular Networking Approach
Source: Foods. 2025 Nov 25;14(23):4042. doi: 10.3390/foods14234042 (PMC12692126; doi:10.3390/foods14234042)
Supplement: Supplementary file 1 [file foods-14-04042-s001.zip › foods-3932829-supplementary.pdf]

Supplementary Material

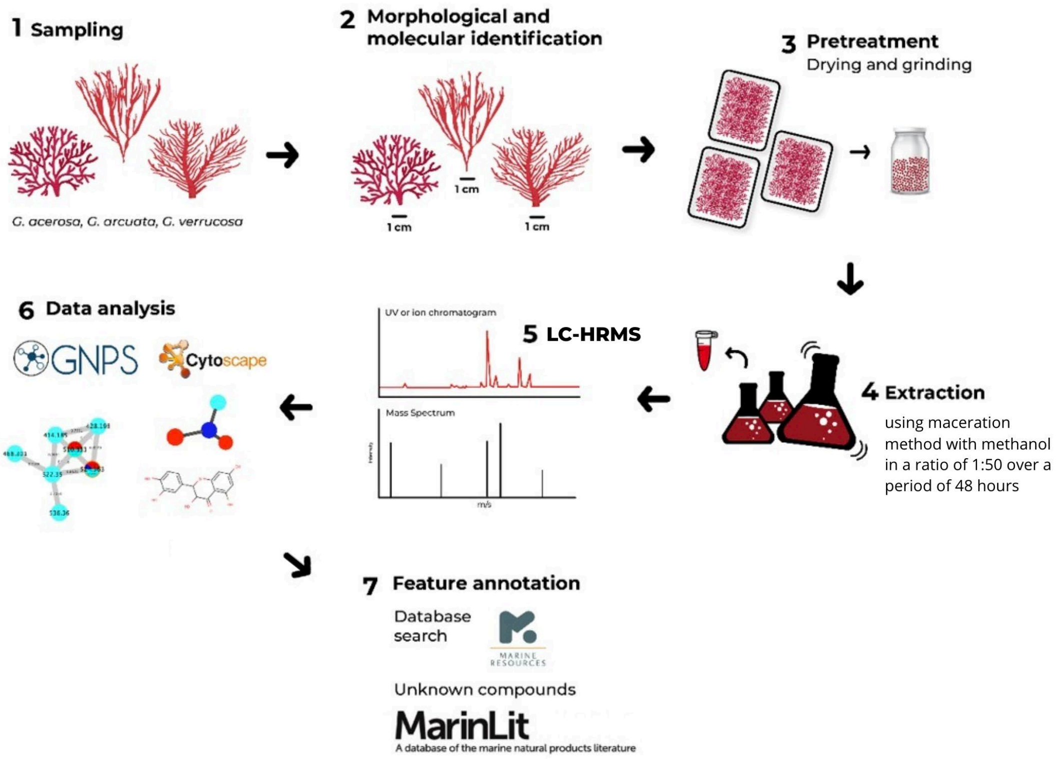

Figure S1. The methodology of metabolomic profiling and molecular networking approach of *Gelidiella acerosa*, *Gracilaria arcuata*, and *Gracilaria verrucosa*
